# Radiation-induced xerostomia and cariogenic dietary habits

**DOI:** 10.1007/s00520-023-08298-x

**Published:** 2024-01-09

**Authors:** Miho Kawashima, Takanori Kawabata, Chikako Ando, Megumi Sakuma, Takashi Aoyama, Hirofumi Ogawa, Tomoya Yokota, Yusuke Onozawa, Takashi Mukaigawa, Tetsuo Nishimura, Keita Mori, Takashi Yurikusa

**Affiliations:** 1 Division of Dentistry and Oral Surgery, Shizuoka Cancer Center, Sunto-Gun, Nagaizumi-Cho, Shizuoka Japan; 2 Clinical Research Center, Shizuoka Cancer Center, Sunto-Gun, Nagaizumi-Cho, Shizuoka Japan; 3 Dietary Department, Shizuoka Cancer Center, Sunto-Gun, Nagaizumi-Cho, Shizuoka Japan; 4 Radiation and Proton Therapy Center, Shizuoka Cancer Center, Sunto-Gun, Nagaizumi-Cho, Shizuoka Japan; 5 Division of Gastrointestinal Oncology, Shizuoka Cancer Center, Sunto-Gun, Nagaizumi-Cho, Shizuoka Japan; 6 Division of Head and Neck Surgery, Shizuoka Cancer Center, Sunto-Gun, Nagaizumi-Cho, Shizuoka Japan

**Keywords:** Xerostomia, Head and neck cancer, Radiotherapy, Dental caries, Dietary habits, Cariogenic

## Abstract

**Purpose:**

Despite the availability of various prevention methods, dental caries continue to be diagnosed in patients receiving head and neck radiotherapy (RT). Since conventional approaches do not evaluate posttreatment alterations in dietary behaviors, we aimed to assess the influence of radiation-induced xerostomia on post-RT cariogenic dietary habits in patients.

**Methods:**

Fifty-seven patients completed the Xerostomia Questionnaire (XQ) and answered questions regarding daily cariogenic food and beverage (CFB) intake, daily tooth brushing, fluoride application, and subjective total taste acuity (STTA). They also underwent evaluations to determine the Simplified Oral Hygiene Index (OHI-S) score, Saxon test score, number of decayed-missing-filled teeth (DMFT), and proportion of DMFT to the test teeth (DMFT rate). Clinical records were searched for information regarding RT modalities, including the median of the mean dose to the parotid glands, days after the completion of RT, submandibular gland resection, whole-neck irradiation, and the DMFT value and rate before RT. The patients were divided into low and high XQ score groups based on the median XQ score of 47.5 for the two sample tests. Univariable and multivariable regression analyses were used to identify independent factors for frequent CFB intake.

**Results:**

Higher XQ scores were associated with a significantly greater frequency of CFB intake (*p* = 0.028*). Regression analysis also identified a higher XQ score (*p* = 0.017*) as an independent risk factor for frequent CFB intake.

**Conclusion:**

Radiation-induced xerostomia increased the frequency of CFB intake.

**Supplementary Information:**

The online version contains supplementary material available at 10.1007/s00520-023-08298-x.

## Introduction

Radiotherapy (RT), often combined with chemotherapy, effectively treats head and neck cancer (HNC) but gives rise to severe oral complications impacting quality of life (QOL). These complications include oral mucositis, xerostomia, dysgeusia, and dental caries; radiation caries present a considerable challenge and have limited proven management options are available, resulting in HNC patients having the highest caries index [1–3].

Dental caries typically arise from a combination of four major factors: 1) host saliva and teeth; 2) cariogenic microflora; 3) substrate or diet; and 4) the exposure time or frequency of dietary intake [4, 5]. RT directly affects the host's salivary glands and tooth structure, with salivary gland dysfunction playing a pivotal role in dental caries development [6]. RT-induced alterations in salivary composition render teeth more susceptible to demineralization, affecting their mineral structure [7, 8]. Reduced salivary flow, a consequence of RT, leads to changes in oral bacterial flora, fostering an increase in cariogenic bacteria in saliva [6, 9]. Maintaining oral hygiene is crucial to minimize the impact of cariogenic microflora [10]. Dietary choices can also contribute to radiation caries, particularly diets rich in soft and carbohydrate-heavy foods [8, 9].

As part of patient education after head and neck RT, patients participate in a comprehensive dental caries prevention program that includes oral hygiene instruction, regular dental visits, and fluoride applications. However, patients’ dietary behaviors have received less attention. To alleviate xerostomia, HNC RT patients tend to frequently consume cariogenic food and beverages (CFB), which can heighten the risk for caries. [11, 12].

This study focused on the frequency of CFB intake, an often overlooked factor in preventing radiation-induced dental caries. Frequent sugar intake has been linked to cariogenic bacteria, and the World Health Organization (WHO) recommends limiting sugar consumption to four times a day for cavity prevention [13–15]. However, the impact of dietary consumption frequency on radiation-induced dental caries has received limited attention. Previous study found no significant effects restriction of sugar-sweetened food on radiation caries.Although it referred to the items of food consumed, it did not account for the frequency of dietary or beverage intake. [16]. Hence, this study aimed to investigate the hypothesis that the high incidence of radiation caries under conventional management is related to the exposure time or frequency of intake of CFBs. We sought to understand the impact of radiation-induced xerostomia on cariogenic dietary behavior changes after RT.

## Materials and methods

### Study participants

From March to July 2015, HNC patients who had received RT were eligible if they (1) had received radical RT with or without chemotherapy at the Shizuoka Cancer Center regardless of the time elapsed since RT completion; (2) were aged 20–80 years; (3) had an Eastern Cooperative Oncology Group Performance Status ≤ 1; (4) had not shown any relapse for at least six months after RT completion; and (5) signed the informed consent form to participate in this study. Patients were excluded based on the following criteria: (1) had no teeth, (2) had tooth hypoplasia, (3) had difficulty chewing and swallowing, (4) had xerostomia induced by other systemic diseases, such as Sjögren’s syndrome, and (5) had psychosis or psychiatric symptoms. Therefore, patients using psychotropic drugs and saliva stimulants were excluded, but there were no specific exclusion criteria for patients using other medications or experiencing medication-induced xerostomia.

### Primary endpoint

The primary endpoint of the study was the difference in the frequency of daily CFB intake between the groups with severe and less severe radiation-induced subjective xerostomia.

### Secondary endpoints

The secondary endpoints were patient-related factors affecting the frequency of CFB intake, xerostomia and dental caries development: salivary flow rates, radiation- and surgery-related factors causing salivary gland disorders, taste acuity, oral hygiene status, preventive behavior for dental caries and dental caries status.

### Data collection

HNC patients who received RT at the study hospital participated in a dental caries prevention program, including oral hygiene guidance, regular dental checkups, and fluoride treatments. Participants signed an informed consent form. Information was collected via interviews and medical record reviews. During a single recall visit, dental hygienists used a questionnaire to analyze CFB intake, subjective xerostomia, and oral hygiene behaviors and determined the saliva flow rate and the Simplified Oral Hygiene Index (OHI-S) score. At the same time, dentists performed oral exams, assessing the number of decayed-missing-filled teeth (DMFT) without performing X-ray. Data on patient characteristics, baseline dental status, fluoride application, and radiation-related factors were extracted from clinical records.

### Definitions of CFBs and non-CFBs

Foods were categorized according to the cariogenic potential index (CPI) derived from Japanese studies [17, 18]. Cariogenic foods included caramel (with the highest CPI of 80), candy (CPI = 60), sugar-sweetened chewing gum (CPI = 50), and chocolate (CPI = 40). As noncariogenic foods, foods with the lowest CPI of 12, including ice cream (CPI = 12), such as rice crackers (CPI = 12), and xylitol chewing gum, were chosen. A thick liquid diet was excluded because it was provided for nutritional needs, not oral comfort.

For beverages, cariogenic potential was determined by pH and adhesive soluble glucan products [19]. Cariogenic beverages encompassed carbonated juice, fruit and vegetable juices, sports drinks, and tea/coffee with sugar. Noncariogenic beverages included water, green tea, and milk; the latter chosen due to its noncariogenic nature and cultural significance in Japan.

### Frequency of CFB intake and the Xerostomia Questionnaire

To ensure precise data collection, daily CFB intake was divided into seven periods to determine the frequency of patients' CFB consumption: (1) late night (snacks or drinks consumed upon waking due to dry mouth); (2) breakfast (meal and drink consumption); (3) after breakfast (snack or drink consumption); (4) lunch (meal and drink consumption); (5) after lunch (snack or drink consumption); (6) dinner (meal and drink consumption); and (7) after dinner (snack or drink consumption). The total frequencies across all periods constituted each patient's daily CFB intake frequency.

### Xerostomia assessment

Subjective xerostomia was determined using the summary score of the Xerostomia Questionnaire (XQ) [20], which comprises eight questions—four each for dryness while eating/speaking and dryness while not eating/speaking. Patients rated each item using an 11-point scale (0–10). Scores ranged from 0–80, with higher scores indicating more severe symptoms. For analysis, the total score was multiplied by 1.25, resulting in a final summary score of 0–100 [12]. Objective xerostomia was determined using the Saxon test [21], which determines the saliva flow rate.

### Salivary gland disorder factors

Clinical records were searched to determine whole-neck irradiation history, radiation modality, days after the completion of RT, mean dose to the parotid glands on the tumor/contralateral side and both sides, and submandibular gland resection history. In cases where the tumor side was indistinguishable (e.g., ethmoid sinus, nasal cavity, or nasopharynx tumors), the mean radiation dose of both parotid glands was considered as the dose for each side.

### Taste acuity

Subjective total taste acuity (STTA) [22, 23] was assessed through interviews.

### Oral hygiene status

The OHI-S score was determined [24].

### Dental caries prevention

Patients reported daily tooth brushing frequency, fluoride toothpaste use, and xylitol gum use. Clinical records were reviewed for regular professional fluoride application.

### Dental caries assessment

Adhering to the Malmö University Oral Health Country/Area Profile Project, wisdom teeth were excluded from the evaluation of DMFT in this study (https://ccarobonapp.mau.se/methods-and-indices/). The DMFT value [25] and DMFT rate, determined by dividing the DMFT value by 28 (the number of test teeth), were assessed. Although previous studies have offered differing opinions regarding whether the mean or median should be used as the representative value [26, 27]; this study defined the median as the representative value.

### Statistical analysis

Wilcoxon's rank-sum test was used to compare continuous variables between the low and high XQ score groups. Fisher's exact test was used for categorical variables. For the frequency of CFB and non-CFB intake, comparisons between the low and high XQ score groups were made, and both were treated as continuous and categorical data to clear their distributions. The frequencies of CFB and non-CFB intake were divided into 3 categories (0, 1 and 2 times per day or more). Univariable and multivariable regression analyses were used to assess the association of dental caries with CFB intake frequency. Nine variables thought to be relevant to CFB intake were analyzed to determine their clinical importance. A negative binomial model was applied to the count data of CFB intake frequency. Risk factors were examined in the multivariable regression analysis using variables with P values of < 0.05 in the univariable regression. All statistical analyses were performed using R version 4.0.3 (R Foundation for Statistical Computing, Vienna, Austria). P values < 0.05 were considered statistically significant.

## Results

### Patient characteristics

A total of 57 participants were included in this study, with 26 (45.6%) having a low XQ score and 31 (54.4%) having a high XQ score. Age, sex, tumor site, and radiation modality was not significantly different between the low and high XQ score groups. There were significant differences in the mean radiation dose to the contralateral side (*p* = 0.007*) and both parotid glands (*p* = 0.002*) and the prevalence of whole-neck irradiation (*p* = 0.008*) (Table 1).

**Table 1:** Patient characteristics

	Overall n = 57	Low XQ score group n = 26 (45.6%)	High XQ score group n = 31 (54.4%)	*p* value
Age (years)	65 (25–80)	64 (29–76)	65 (29–78)	0.784
Sex				
Male	45 (78.9%)	20 (76.9%)	25 (80.6%)	0.755
Female	12 (21.1%)	6 (23.1%)	6 (19.4%)	
Tumor site				
Maxillary/ethmoid sinus, nasal cavity or nasopharynx	17 (29.8%)	9 (34.6%)	8 (25.8%)	0.240
Oral cavity or oropharynx	30 (52.6%)	12 (46.2%)	18 (58.1%)	
Hypopharynx or larynx	5 (8.8%)	1 (3.8%)	4 (12.9%)	
Parotid or submandibular gland	5 (8.8%)	4 (15.4%)	1 (3.2%)	
Radiation modality				
Conventional RT	47 (82.5%)	20 (76.9%)	27 (87.1%)	0.486
IMRT	10 (17.5%)	6 (23.1%)	4 (12.9%)	
Median of the mean dose to the parotid glands (Gy)				
Tumor side	44.7 (6.3–70.3)	42.2 (6.3–70.3)	48.0 (14.6–70.0)	0.130
Contralateral side	37.3 (0.3–68.4)	12.3 (0.3–52.0)	38.6 (1.2–68.4)	0.007*
Both parotid glands	38.4 (0.5–69.2)	29.3 (0.5–52.3)	41.4 (2.2–69.2)	0.002*
Days after the completion of RT	863(192–4141)	769 (192–4141)	863 (237–3999)	0.753
Submandibular gland resection	2 (42.1%)	12 (46.2%)	12 (38.7%)	0.601
Concomitant chemotherapy	37 (64.9%)	15 (57.7%)	22 (71.0%)	0.405
RT as adjuvant therapy	28 (49.1%)	15 (57.7%)	13 (41.9%)	0.292
Whole-neck irradiation	29 (50.9%)	8 (30.8%)	21 (67.7%)	0.008^*^

^*^p value < 0.05; IMRT, intensity-modulated radiation therapy; RT, radiotherapy

### Xerostomia and CFB intake frequency

For the primary endpoint, significant distinctions emerged in the overall frequency of CFB intake (*p* = 0.028*) and the consumption of coffee or tea with sugar (*p* = 0.046*) between the low and high XQ score groups. Conversely, no notable differences were found in the total frequency of non-CFB intake between groups. The XQ score significantly differed between the two groups. The overall median XQ scores were 47.5 (0–95) and 11.25 (0–46.25) for the low XQ score group and 65 (47.5–95) for the high XQ score group (*p* < 0.001*) (Table 2).

**Table 2:** Frequency of CFB and non-CFB intake at different XQ levels

	Median frequency among all patients	Median frequency in the low XQ score group (XQ score < 47.5)	Median frequency in the high XQ score group (XQ score ≥ 47.5)	*p* value
XQ score	47.5 (0–95)	11.25 (0–46.25)	65 (47.5–95)	< 0.001^*^
Total CFB intake	1 (0–18)	0.5 (0–8)	2 (0–18)	0.028^*^
Chewing gum	0 (0–3)	0 (0–3)	0 (0–3)	0.204
Chocolate	0 (0–3)	0 (0–3)	0 (0–2)	1.000
Sugar-sweetened candy	0 (0–10)	0 (0–2)	0 (0–10)	0.317
Caramel	0(0–6)	0 (0–0)	0 (0–6)	0.494
Carbonated juice	0 (0–3)	0 (0–1)	0 (0–3)	1.000
Sports drink	0 (0–3)	0 (0–2)	0 (0–3)	0.615
Fruit or vegetable juice	0 (0–3)	0 (0–2)	0 (0–3)	0.413
Coffee or tea with sugar	0 (0–18)	0 (0–3)	1(0–18)	0.046*
CFB intake (in intervals)				
Late night	0 (0–4)	0 (0–0)	0 (0–4)	1.000
At breakfast	0 (0–2)	0 (0–1)	0 (0–2)	0.581
After breakfast	0 (0–6)	0 (0–3)	0 (0–6)	0.643
At lunch	1 (0–2)	0 (0–1)	0 (0–2)	0.639
After lunch	0 (0–12)	0 (0–3)	1 (0–12)	0.008^*^
At dinner	0 (0–4)	0 (0–4)	0 (0–1)	1.000
After dinner	0 (0–6)	0 (0–1)	0 (0–6)	0.529
Total non-CFB intake	9 (0–47)	7 (0–30)	9 (0–47)	0.437
Rice crackers	0 (0–4)	0 (0–3)	0 (0–4)	0.579
Ice cream	0 (0–2)	0 (0–2)	0 (0–2)	0.480
Milk	1 (0–4)	0.5 (0–4)	1.0 (0–3)	0.706
Bottled green tea	0 (0–10)	0 (0–3)	0 (0–10)	0.848
Brewed green tea	3 (0–25)	3 (0–26)	2 (0–20)	0.246
Bottled water	2 (0–45)	2 (0–18)	2 (1–45)	0.239
Xylitol gum	0 (0–6)	0 (0–6)	0 (0–2)	0.449
Non-CFB intake (in intervals)				
Late night	0 (0–4)	0 (0–2)	0 (0–4)	0.948
At breakfast	1 (0–10)	2 (0–3)	1 (0–10)	0.884
After breakfast	1 (0–15)	1 (0–8)	1 (0–15)	0.251
At lunch	1 (0–10)	1 (0–3)	1 (0–10)	0.600
After lunch	2 (0–15)	2 (0–8)	2 (0–15)	0.445
At dinner	1 (0–3)	1 (0–3)	1 (0–3)	0.389
After dinner	1 (0–15)	1 (0–9)	1 (0–15)	0.951

^*^p value < 0.05

Overall, 61.4% of the patients consumed CFBs daily, and the proportion was 50.0% in the low XQ score group and 74.2% in the high XQ score group (*p* = 0.10). The odds ratio (OR) for CFB intake in the high XQ score group was 2.88 (95% CI: 0.94–8.75, *p* = 0.063). The high XQ score group showed a higher proportion of patients consuming coffee or tea with sugar (54.8%) than the low XQ score group (26.9%, *p* = 0.037*), with an OR of 3.30 (95% CI: 1.08–10.09). Chewing gum consumption was the lowest overall (3.5%) and tended to be higher in the low XQ score group (7.7%) than in high XQ score group (0.0%, p = 0.20). Fifty-five (96.5%) patients consumed non-CFB items, with bottled water (73.7%) being the most common, followed by freshly brewed green tea (70.2%) and milk (50.9%). Xylitol gum was consumed the least (8.8%), with a higher proportion of patients in the low XQ score group (11.5%) than in the high XQ score group (6.5%, *p* = 0.651). No statistically significant differences were found between the low and high XQ score groups for non-CFB items (Table 3).

**Table 3:** The proportions of patients reporting daily intake of CFB and non-CFB items with different XQ scores

	Proportion (%) of all patients	Proportion (%) in the low XQ score group < 47.5)	Proportion (%) in the high XQ score group XQ ≥ 47.5)	p value	OR (95% CI)	*p* value
Total CFB intake	35(61.4%)	13 (50.0%)	23 (74.2%)	0.10	2.88 (0.94–8.75)	0.063
Chewing gum	2 (3.5%)	2 (7.7%)	0(0.0%)	0.20	- (0-inf)	0.995
Chocolate	10(17.5%)	5 (19.2%)	6 (19.4%)	1.00	1.000 (0.273.78)	0.991
Sugar-sweetened candy	7 (12.3%)	2 (7.7%)	5 (16.1%)	0.436	2.31 (0.41–13.0)	0.344
Caramel	2 (3.5%)	0 (0.0%)	2 (6.5%)	0.495	- (0-inf)	0.996
Carbonated juice	4 (7.0%)	2 (7.7%)	2 (6.5%)	1.000	0.83 (0.11–6.21)	0.855
Sports drink	8 (14.0%)	3 (11.5%)	5 (16.1%)	0.715	1.47 (0.32–6.86)	0.621
Fruit or vegetable juice	12 (21.1%)	4 (15.4%)	8 (25.8%)	0.516	1.91 (0.50–7.27)	0.341
Coffee or tea with sugar	24 (42.1%)	7 (26.9%)	17 (54.8%)	0.058	3.30 (1.08–10.09)	0.037*
Total Non-CFB intake	55 (96.5%)	25 (96.2%)	30 (96.8%)	1.00	1.20 (0.07–20.18)	0.899
Rice crackers	13 (22.8%)	7 (26.9%)	6 (19.4%)	0.541	0.65(0.19–2.26)	0.499
Ice cream	11 (19.3%)	6 (23.1%)	5 (16.1%)	0.524	0.64(0.17–2.41)	0.510
Milk	29 (50.9%)	13(50.0%)	16 (51.6%)	1.000	1.07(0.38–3.03)	0.903
Bottled green tea	16 (28.1%)	7 (26.9%)	9 (29.0%)	1.000	1.11(0.35–3.55)	0.860
Brewed green tea	40 (70.2%)	20 (76.9%)	20 (64.5%)	0.389	0.55 (0.17–1.76)	0.310
Bottled water	42 (73.7%)	17 (65.4%)	25 (80.6%)	0.236	2.2(0.66–7.34)	0.197
Xylitol gum	5 (8.8%)	3 (11.5%)	2 (6.5%)	0.651	0.53(0.08–3.43)	0.504

^*^p value < 0.05

inf, infinity; OR, odds ratio; CFB, Cariogenic food and beverage; non-CFB, noncariogenic food and beverage; Proportion (%), the percentage of patients in each group who consumed each item at least once per day

**Fig. 1:**
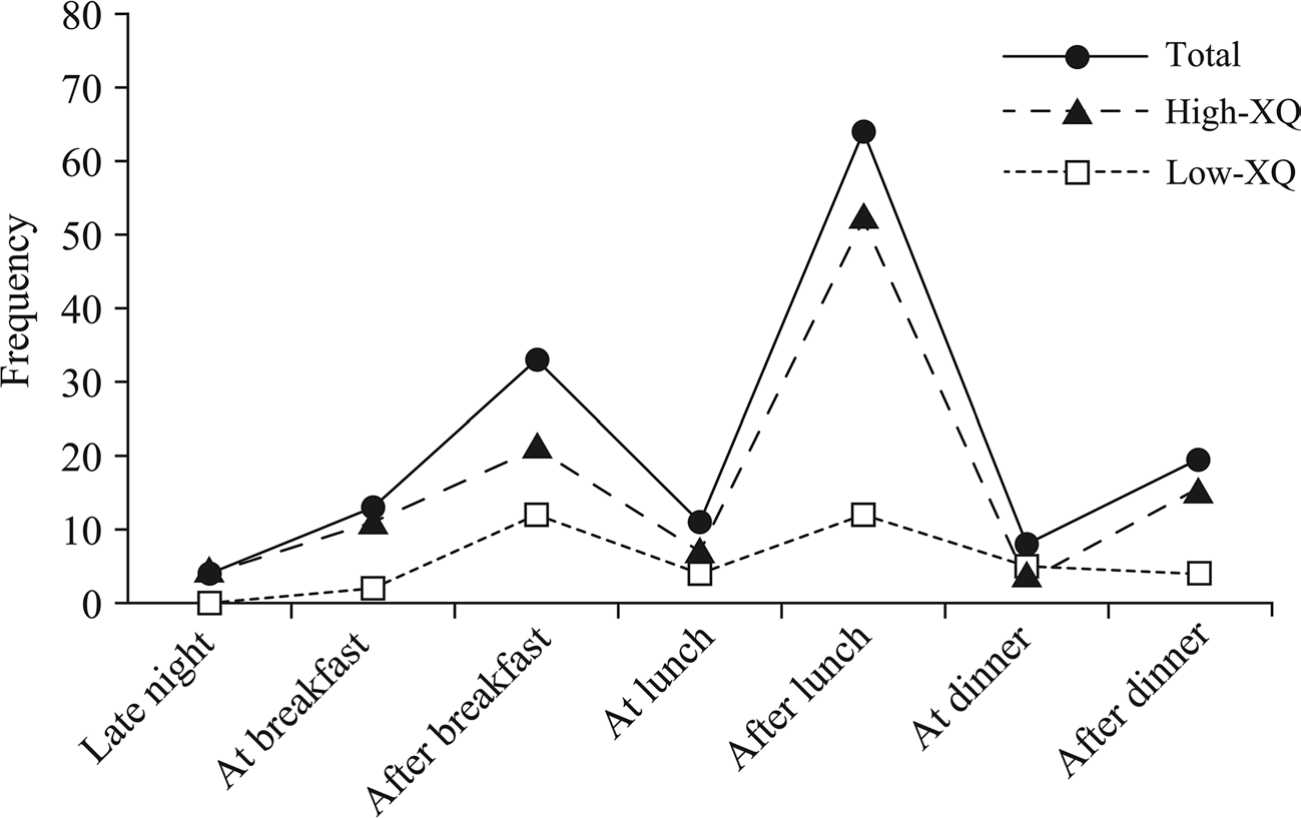
Frequency of cariogenic food and beverage (CFB) intake at different times of the day; XQ, Xerostomia Questionnaire

When comparing CFB and non-CFB intake by categories (0, 1, and 2 times per day or more), there were significant differences in total daily frequency. Noteworthy temporal variations were identified, particularly in the frequency of CFB intake after lunch (*p* = 0.008*) (Table 2) (Fig. 1). Regardless of whether the frequency data were treated as categorical variables or continuous variables, the results indicated significant differences in the identified timeframes consistently. Detailed information about the frequency distribution of categories can be found in Table S1 (Online Resource 1).

A strong association between a high XQ score and increased CFB intake frequency was observed (RR = 2.43, *p* = 0.017*) (Table 4). However, age, sex, taste acuity, salivary flow rate, whole-neck RT history, radiation dose to the parotid glands, tumor site, and days after the completion of RT showed no significant influence on CFB intake frequency. Univariate regression results indicated no other statistically significant risk factors, making multivariate analysis unnecessary.

**Table 4:** Negative binomial regression for frequent CFB intake

	Univariable analysis		
	RR	95% CI	*p* value
Age	1.00	0.97–1.03	0.874
Sex			
Male	Ref		
Female	2.31	0.97–5.44	0.054
XQ			
Low XQ score	Ref		
High XQ score	2.43	1.17–5.05	0.017^*^
STTA	1.11	0.78–1.60	0.559
Saxon test (g)	1.19	0.76–1.88	0.440
Whole-neck RT	0.82	0.39–1.75	0.614
Mean dose to the parotid gland (Gy)			
Tumor side	0.98	0.96–1.01	0.208
Contralateral side	0.98	0.96–1.01	0.336
Both parotid glands	0.99	0.97–1.01	0.278
Tumor site			
Hypopharynx or Larynx	Ref		
Oral cavity or Oropharynx	1.69	0.42–6.75	0.461
Maxillary/ethmoid sinus, Nasal cavity or nasopharynx	1.54	0.36–6.60	0.564
Parotid or submandibular gland	0.56	0.08–3.80	0.550
Days after the completion of RT	1.00	1.00–1.00	0.634

CFB, cariogenic food and beverage; CI, confidence interval; Ref: reference category (RR = 1.00); RR, relative risk; RT, radiotherapy; XQ, Xerostomia Questionnaire

^*^p value < 0.05

Regarding the secondary endpoints, participants with high XQ scores had lower salivary flow rates (Saxon test, *p* = 0.028*) and higher subjective taste acuity (STTA, *p* = 0.001*) (Table 5). There were no significant differences in oral hygiene practices or dental caries parameters between the low and high XQ score groups (Table 5).

**Table 5:** Dental caries, oral hygiene status, and other factors related to xerostomia

	Overall n = 57	Low XQ score group n = 26 (45.6%)	High XQ score group n = 31 (54.4%)	*p* value
Saxon test (g)	0.71 (0.03–4.77)	0.81 (0.12–4.77)	0.57 (0.03–2.63)	0.028^*^
STTA	1 (0–4)	1 (0–2)	1 (0–4)	0.001^*^
Daily frequency of tooth brushing	3 (1–4)	3 (1–4)	3 (1–4)	0.270
OHI-S	0.66 (0–2.33)	0.66 (0–1.66)	0.5 (0–2.33)	0.445
Use of fluoride toothpaste	33 (57.9%)	12 (46.2%)	21 (67.7%)	0.711
Topical fluoride application	47 (82.5%)	21 (80.8%)	25 (80.6%)	1.000
Use of Xylitol gum	5 (8.8%)	3 (11.5%)	2 (6.5%)	0.651
DMFT value				
before RT	15 (0–28)	14 (0–28)	15 (0–28)	0.986
after RT	19 (0–28)	17 (3–28)	22 (0–28)	0.244
DMFT rate				
before RT	53.6% (0–100%)	50.0% (0–100%)	53.6% (0–100%)	0.990
after RT	67.9% (0–100%)	60.7% (11–100%)	78.6% (0–100%)	0.248
Increment of				
DMFT value	1 (0–18)	1 (0–13)	1 (0–18)	0.270
DMFT rate	3.6% (0–64%)	3.6% (0–46.4%)	3.6% (0–64.3%)	0.286

^*^p value < 0.05

DMFT, decayed-missing-filled teeth; OHI-S, Simplified Oral Hygiene Index; RT, radiotherapy; STTA, subjective total taste acuity; XQ, Xerostomia Questionnaire

## Discussion

This study investigated the impact of radiation-induced xerostomia on HNC patients’ daily eating and drinking habits after RT and, in particular, the relationship between the frequency of CFB intake and subjective xerostomia scores. Regarding the frequency of eating and drinking, we focused not only on meal intake but also on snack and fluid intake. The study indicated that subjective xerostomia was associated with salivary flow rate. In other words, the lower the salivary flow rate was, the stronger subjective xerostomia the patients would demonstrate. Severe subjective xerostomia was also significantly associated with taste acuity and higher daily CFB intake, especially coffee or tea with sugar. However, although salivary flow rates and taste acuity differed between the xerostomia groups, the univariate analysis emphasized that subjective xerostomia was the main risk factor for frequent CFB intake, surpassing the impact of objective xerostomia or other variables.

The definition of CFBs was based on Japanese studies [17, 19] but was also consistent with international evaluations of cariogenicity. Reported cariogenic foods and drinks include chewing gum [28], chocolate [29, 30], candy [28], caramel [28], carbonated juices [28, 31–33], sports drinks [32], fruit and vegetable juices [28, 31], and coffee or tea with sugar [28, 29]. Milk [30, 31, 34, 35] and tea without sugar [35] were considered noncariogenic. Classifying ice cream as a non-CFB is controversial; some studies have shown that ice cream is a food that can cause tooth decay [28–30]. These variations may be due to differences in sugar amounts among countries. Ice cream has a CPI of 12 [17, 19], indicating minimal cariogenic potential, but is not necessarily noncariogenic. Similarly, one study classified fruit juice as noncariogenic [30].

The participants in this study were a population with subjective and objective severe xerostomia, with a median XQ score of 47.5 and a median Saxon test score of 0.71 g. In a previous report, the approximate XQ score was 40 [20], and the Saxon test diagnostic criterion for xerostomia was 2.0 g [21]. This study divided patients into two groups based on the XQ score and found significant differences in the XQ score and salivary flow rate. The difference in xerostomia between the two groups was attributed to the total radiation dose, especially "the mean dose" to the contralateral side and both parotid glands, and whole-neck irradiation.

After RT, HNC patients may choose CFBs over non-CFBs for intraoral comfort, but this is often due to lack of awareness about cariogenicity. Because sweet taste improves early in the post-RT taste recovery process [36], HNC patients may develop a preference for sweet drinks such as coffee or tea with sugar after RT. There was a significant difference in taste acuity between the low-XQ and the high- XQ groups in this study. However, taste acuity was not a significant risk factor for frequent CFB intake in univariate analysis, possibly due to its focus on evaluating taste changes before and after treatment rather than specifically addressing individual taste sensitivities. The after-lunch period is characterized by tea-time habits and includes various motivations, such as the desire for intraoral comfort and tea-time snacks and drinks, resulting in an increased frequency of CFB intake. HNC patients with severe xerostomia should avoid chewing gum, whether sugar-based or xylitol-based, after RT due to decreased salivary secretion. This result suggests that xylitol gum may not be effective in preventing radiation-induced dental caries in HNC patients after RT.

High dental caries morbidity was observed in both participants with low XQ and high XQ scores. Despite diligent oral hygiene habits, the prevalence of dental caries was high, with a median DMFT value of 19.0, exceeding a previously reported value of 17.01 [3]. This indicates that current caries prevention measures, such as oral hygiene management and topical fluoride application, are inadequate. On the other hand, the DMFT value may not precisely measure radiation caries and may not result in significant differences between groups in high-prevalence populations. The nature of the DMFT value, considering a tooth with both a carious lesion and filling as only decayed, may have underestimate caries prevalence. These factors may contribute to the lack of a significant differences in caries prevalence between the groups.

In radiation-induced caries management, it is necessary to focus on the fact that HNC patients with severe xerostomia after RT tend to increase their CFB intake. Patient education is important to encourage tooth-friendly food and drink choices and discourage the consumption of CFBs for intraoral comfort. To limit CFB intake through snacks, it is recommended to follow the WHO sugar intake guidelines (up to 4 servings per day) [37], and future studies should consider specific recommendations for patients with radiation-induced xerostomia. For patients seeking sweetness, alternative sweeteners are recommended, such as xylitol candy or xylitol gum, which have shown efficacy in preventing tooth decay [38] and increasing saliva production [39]. However, as shown in this study, severe xerostomia after RT or malocclusion due to radiation-induced caries can make xylitol gum difficult to utilize. Regarding other alternative approaches to improve xerostomia, studies have reported that approximately 50% of patients respond positively to pilocarpine hydrochloride, although side effects must be considered [40]. Because dentin and enamel have different pH thresholds for demineralization, the acidity of bottled beverages should also be accounted for[33], especially in elderly patients with gingival recession [6, 33]. Some patients have difficulty swallowing and chewing due to cancer treatment, resulting in the intake of CFBs, which are often found in soft or thick liquid foods [41–43]. Therefore, future studies should recognize the risks associated with the frequency of CFB intake and the influence of CFBs on the development of radiation-induced caries and develop caries prevention strategies for HNC patients after RT.

## Limitations

The limitations of this study include the relatively small sample size, potential selection bias in CFB/non-CFB discrimination, and the inability to directly assess the impact of CFB intake on caries development. This study also lacked detailed information on patients' dietary habits before RT, hindering a comprehensive understanding of factors influencing the development of radiation caries. Future studies should include daily monitoring of the entire diet to elucidate the relationship among xerostomia, dietary choices, and radiation-induced caries.

## Conclusion

This study revealed that radiation-induced xerostomia causes frequent CFB intake. Salivary gland dysfunction may increase the risk of radiation caries by directly affecting teeth and indirectly encouraging cariogenic dietary habits due to subjective xerostomia.

### Supplementary Information

Below is the link to the electronic supplementary material.

Supplementary file1 (XLSX 16 KB)

## Data Availability

The data that support the findings of this study are available from the corresponding author, T. Yurikusa, upon reasonable request.
